# Capsaicin triggers immunogenic PEL cell death, stimulates DCs and reverts PEL-induced immune suppression

**DOI:** 10.18632/oncotarget.4911

**Published:** 2015-08-18

**Authors:** Marisa Granato, Maria Saveria Gilardini Montani, Mariarosari Filardi, Alberto Faggioni, Mara Cirone

**Affiliations:** ^1^ Department of Experimental Medicine, Sapienza University of Rome. 00161 Rome, Italy

**Keywords:** capsaicin, Immunogenic cell death, STAT3, DCs, PEL

## Abstract

Capsaicin, the pungent alkaloid of red pepper has been extensively studied for its many properties, especially the anti-inflammatory and anti-oxidant ones. It binds to vanilloid receptor 1, although it has been reported to be able to mediate some effects independently of its receptor. Another important property of Capsaicin is the anticancer activity against highly malignant tumors, alone or in combination with other chemotherapeutic agents. In this study, we found that Capsaicin induced an apoptotic cell death in PEL cells correlated with the inhibition of STAT3. STAT3 pathway, constitutively activated in PEL cells, is essential for their survival. By STAT3 de-phosphorylation, Capsaicin reduced the Mcl-1 expression level and this could represent one of the underlying mechanisms leading to the Capsaicin-mediated cell death and autophagy induction. Next, by pharmacological or genetic inhibition, we found that autophagy played a pro-survival role, suggesting that its inhibition could be exploited to increase the Capsaicin cytotoxic effect against PEL cells. Finally, we show that Capsaicin induced DAMP exposure, as for an immunogenic cell death, directly promoted DC activation and, more importantly, that it counteracted the immune-suppression, in terms of DC differentiation, mediated by the PEL released factors.

## INTRODUCTION

Capsaicin (*trans*-8-methyl-N-vanillyl-6-nonenamide) is the principal component of hot peppers, plants from the genus *Capsicum,* member of *Solanaceae* family. Capsaicin has been shown to exert many positive effects on cardiovascular and gastrointestinal systems and has also been employed in pain relief, weight loss and cancer prevention [1]. Besides that, Capsaicin has an anticancer effect against several solid [2–5] and hematological tumors [6]. Among them, Capsaicin has been shown to suppress cell proliferation and trigger apoptosis of Multiple Myeloma (MM) cells, by reducing STAT3 phosphorylation and activation [7]. The activation of STAT3 pathway, mainly due to the effect of tumor-released factors, plays indeed a critical role in cell survival and chemo-resistance of MM as well as several other tumor cells [8–10]. STAT3 is constitutively activated also in Primary Effusion Lymphoma (PEL) cells and its inhibition leads to apoptotic cell death [11, 12]. Besides STAT3, PEL cells relay on the constitutive activation of other pathways for their survival [13, 14]. In this study, we investigated whether Capsaicin would affect PEL cell survival and reduce the STAT3 constitutive phosphorylation. Moreover, we explored whether Capsaicin would also induce autophagy in PEL cells and its role on cell viability. Previous studies have shown that Capsaicin can induce autophagy either as a pro-death [15] or as a pro-survival mechanism [16, 17]. The expression level of molecules belonging to Bcl-2 family, such as Mcl-1, have been reported to be influenced by the level of STAT3 phosphorylation [18, 19] and regulate both apoptosis and autophagy [20]. Thus, we next evaluated the level of expression of Mcl-1 in PEL cells treated with Capsaicin, in comparison with cells treated with AG490 STAT3 inhibitor, to investigate whether STAT3 inhibition could be a possible underlying mechanism influencing apoptosis and autophagy in PEL cells treated with Capsaicin. Besides successfully killing tumor cells, Capsaicin has been reported to have also immune-modulating properties, being able to activate DCs through the vanilloid receptor 1 (VR1) [21] Moreover, Capsaicin has given promising results in the activation of antitumor immune response also *in vivo*, in animal models, [22] although contradictory studies showing a negative effect of Capsaicin on DCs have also been reported [23]. DCs play a pivotal role in the priming of immune response against novel antigens and their activation is essential for the complete tumor eradication, especially in the course of the chemotherapeutic treatments [24, 25]. Thus, we next investigated the effect of Capsaicin (at the same dose used to kill PEL cells) on monocyte-derived DCs, and compared its effect to that obtained by using lipolysaccharide (LPS), the classical DC activator. Another important effect of Capsaicin, observed in this study was its capacity to cause a pre-apoptotic DAMP surface exposure in PEL cells that may also indirectly lead to DC activation. PEL cells release a variety of cytokines and soluble factors that, besides promoting tumor cell survival, impair monocyte differentiation into functional DCs [26]. So, we finally evaluated if Capsaicin would be able to counteract such immune-suppressive effect and rescue monocyte differentiation into DCs. A treatment like Capsaicin, having the capacity to induce an immunogenic cell death in tumor cells, to activate the immune system and to counteract the tumor-mediated immune-suppression may allow to obtain the goals of an ideal anticancer therapy.

## RESULTS

### Capsaicin induces an immunogenic apoptotic cell death in PEL cells

**Figure 1:**
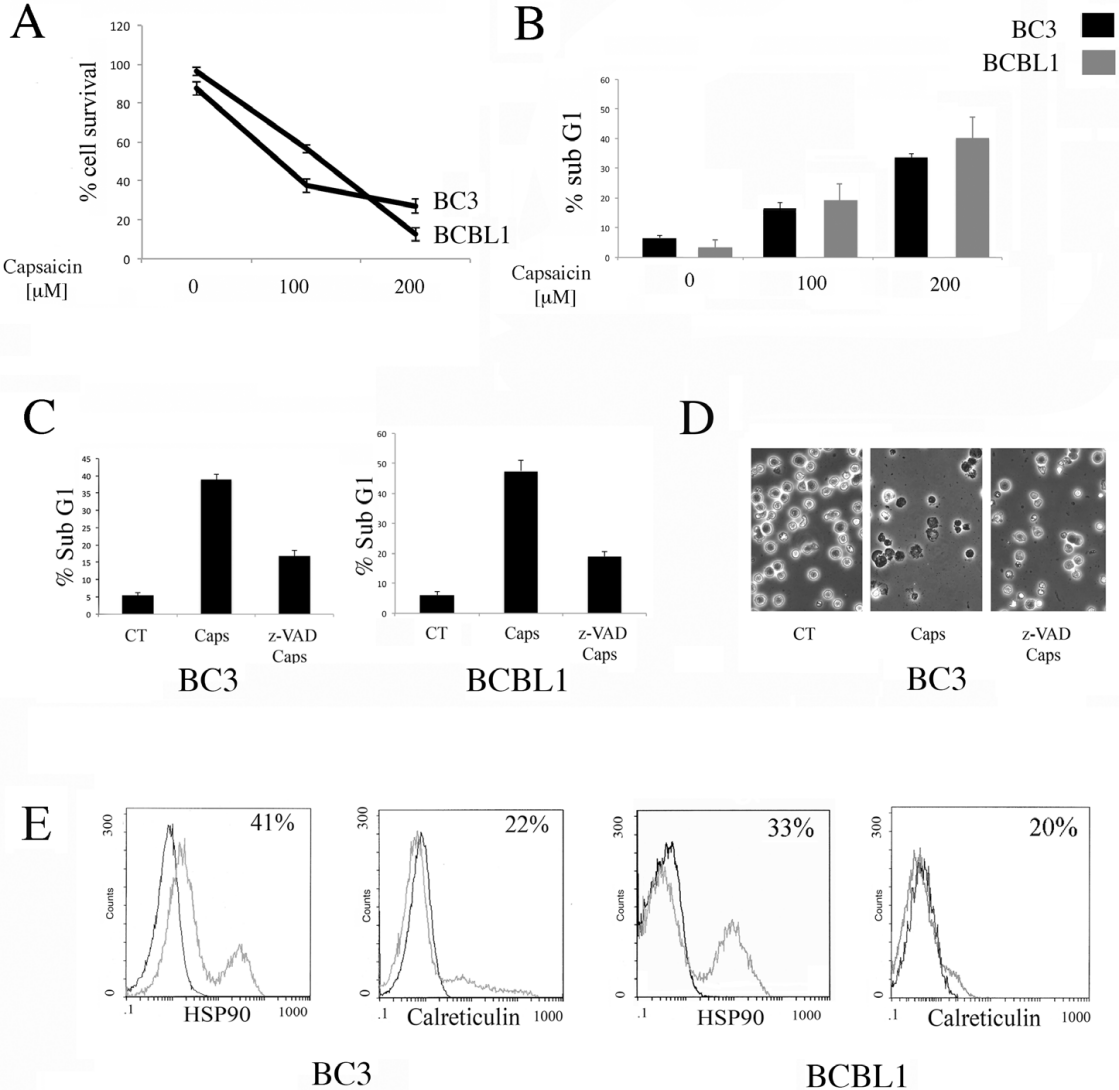
Capsaicin induces an immunogenic apoptosis in PEL cells **A.** Capsaicin-induced loss of viability in PEL cell lines is dose-dependent. BC3 and BCBL1 were treated for 24 hours with different Capaicin concentrations (100 and 200 μM). Cells were counted by trypan blue exclusion and mean of the percentage of cell survival plus SD of three independent experiments is indicated; **B.** BC3 and BCBL1 cells were treated with Capsaicin (100 and 200 μM) and the effect of the drugs on nuclear fragmentation was also evaluated (Sub-G1 phase), representing the apoptotic cells. Mean of the percentage of sub-G1 cells plus SD of three independent experiments is indicated; **C.** The reduction of the percentage of the Sub-G1 cells obtained with Z-VAD-fmk (z-VAD) pre-treatment at 50 μM is also shown. Mean of the percentage of positive cells plus SD of three independent experiments is indicated; **D.** The reduction of the BC3 cell death obtained with Z-VAD-fmk (z-VAD) pre-treatment, at 50 μM, is evidenced by optical microscopic observation; **E.** Induction of HSP90 and CRT translocation on BC3 and BCBL1 cell surface induced by Capsaicin after 12 hours of treatment is shown. Percentage of positive cells of a representative experiment is shown.

BC3 and BCBL1 PEL cells were treated with two different doses of Capsaicin (100 and 200 μM) for 24 hours and its effect on cell survival was assessed by trypan-blue exclusion assay. We found that Capsaicin reduced cell survival in a dose-dependent fashion, in both PEL cell lines (Figure 1A). We then found that Capsaicin induced an increase of the sub-G1 events (Figure 1B), suggesting the occurrence of an apoptotic cell death. To further confirm the apoptotic cell death, PEL cells were treated with Capsaicin in the presence or in the absence of z-VAD-fmk pan-caspase inhibitor z-VAD. As shown in Figure 1C and 1D, z-VAD-fmk was able to partially revert the percentage of sub-G1 cells as well as the cell death, as indicated by optic microscopic observations. From the immunogenic point of view, an apoptotic cell death can be immunogenic or not immunogenic, depending on its ability to induce the surface exposure of Damage Associated Molecular Patterns (DAMPs) such as HSP90 and Calreticulin, that in turn activate the immune system. So, we next investigated whether Capsaicin would induce a pre-apoptotic DAMP exposure and found that HSP90 and Calreticulin were detected on the cell surface of PEL cells treated with Capsaicin for 12 hours (Figure 1E). All together these results indicate that Capsaicin induces an immunogenic apoptosis in PEL cells.

### Capsaicin inhibits STAT3 constitutive activation in PEL cells

**Figure 2:**
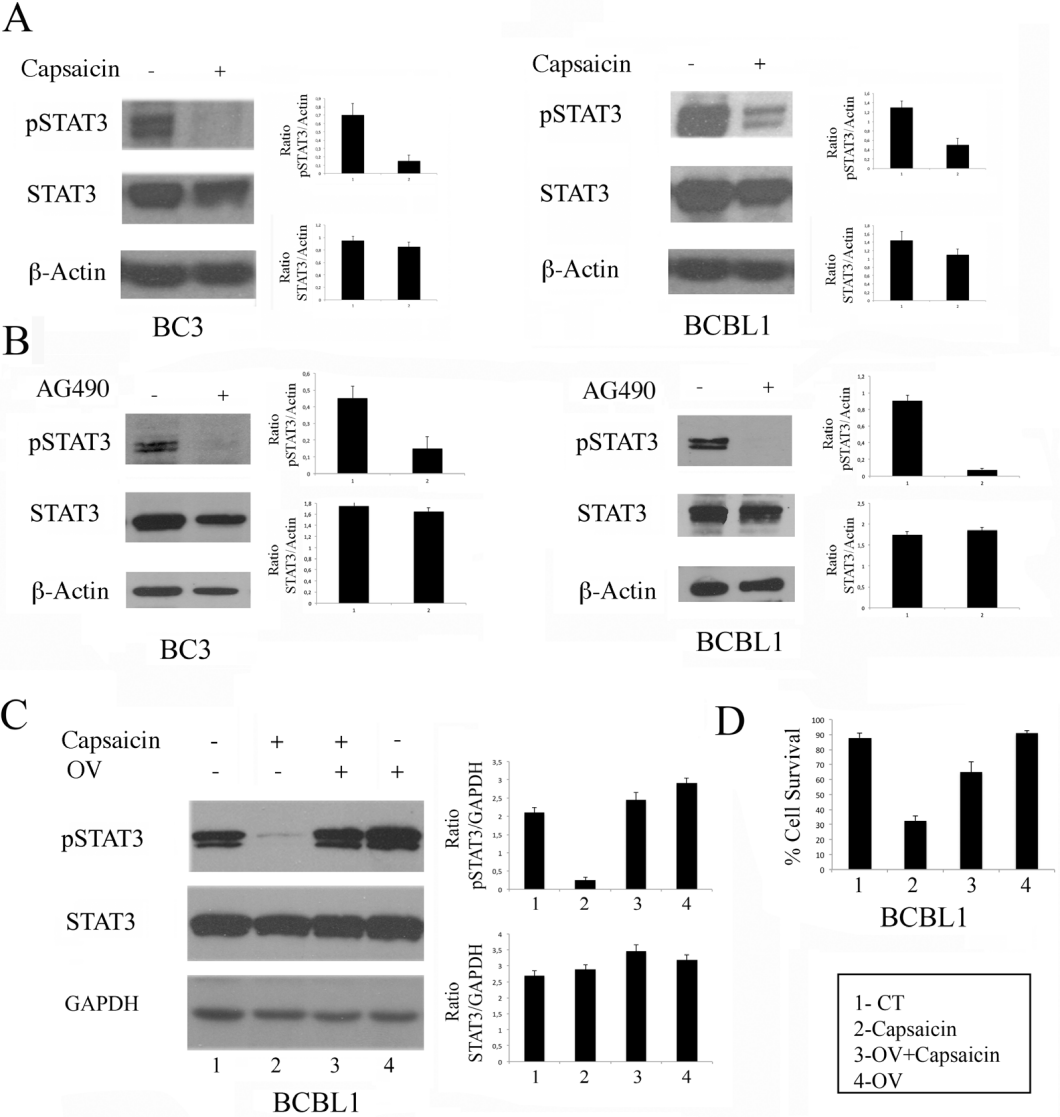
The reduction of STAT3 phosphorylation is involved in Capsaicin-mediated cell death **A.** BC3 and BCBL1 were treated for 24 hours with Capaicin (200 μM) and STAT3 tyrosine phosphorylation was evaluated by western blot analysis. Total STAT3 and β-Actin were included as control. A representative experiment is shown and the mean plus SD of the densitometric analysis of the specific proteins on β-Actin of three independent experiments is also reported; **B.** BC3 and BCBL1 were treated for 24 hours with AG490 (100 μM) and STAT3 tyrosine phosphorylation was evaluated by western blot analysis. Total STAT3 and Actin were included as control. A representative experiment is shown and the mean plus SD of the densitometric analysis of the specific proteins on β-Actin of three independent experiments is also reported; **C.** BCBL1 PEL cells were treated for 24 hours with Capsaicin (200 μM) in the presence or in the absence of orthovanadate (OV) (100 μM) and STAT3 tyrosine phosphorylation was evaluated by western blot analysis. Total STAT3 and GAPDH were included as control. Mean plus SD of the densitometric analysis of the specific proteins on GAPDH of three independent experiments is also reported; **D.** Cell viability of BCBL1 PEL cells treated for 24 hours with Capaicin (200 μM) in the presence or in the absence of OV (100 μM). Cells were counted by trypan blue exclusion and mean of the percentage of cell survival plus SD of three independent experiments is shown.

Since PEL cells relay on the constitutive activation of STAT3 for their survival, we next investigated if Capsaicin impairment of PEL cell survival would correlate with a reduction of the STAT3 phosphorylation, as previously demonstrated in MM [7]. At this aim, both BC3 and BCBL1 cells were treated with Capsaicin for 24 hours and, as shown in Figure 2A, Capsaicin (200 μM) strongly suppressed STAT3 tyrosine phosphorylation. The STAT3 de-phosphorylating effect mediated by Capsaicin was similar to the effect obtained by treating PEL cells with the tyrosine kinase inhibitor tyrphostin AG490 (Figure 2B). Capsaicin-induced STAT3 de-phosphorylation was reverted by the broad-acting tyrosine phosphatase inhibitor sodium orthovanadate (OV) [27] (Figure 2C), indicating that tyrosine phosphatases play a major role in capsaicin-mediated STAT3 de-phosphorylation. OV treatment also reduced the cytotoxic effect mediated by Capsaicin against BCBL1 PEL cells (Figure 2D), suggesting that STAT3 de-phosphorylation was involved in the Capsaicin-mediated cytotoxic effect.

### Capsaicin induces caspase-dependent Mcl-1 cleavage and cell death similarly to AG490 STAT3 inhibitor

**Figure 3:**
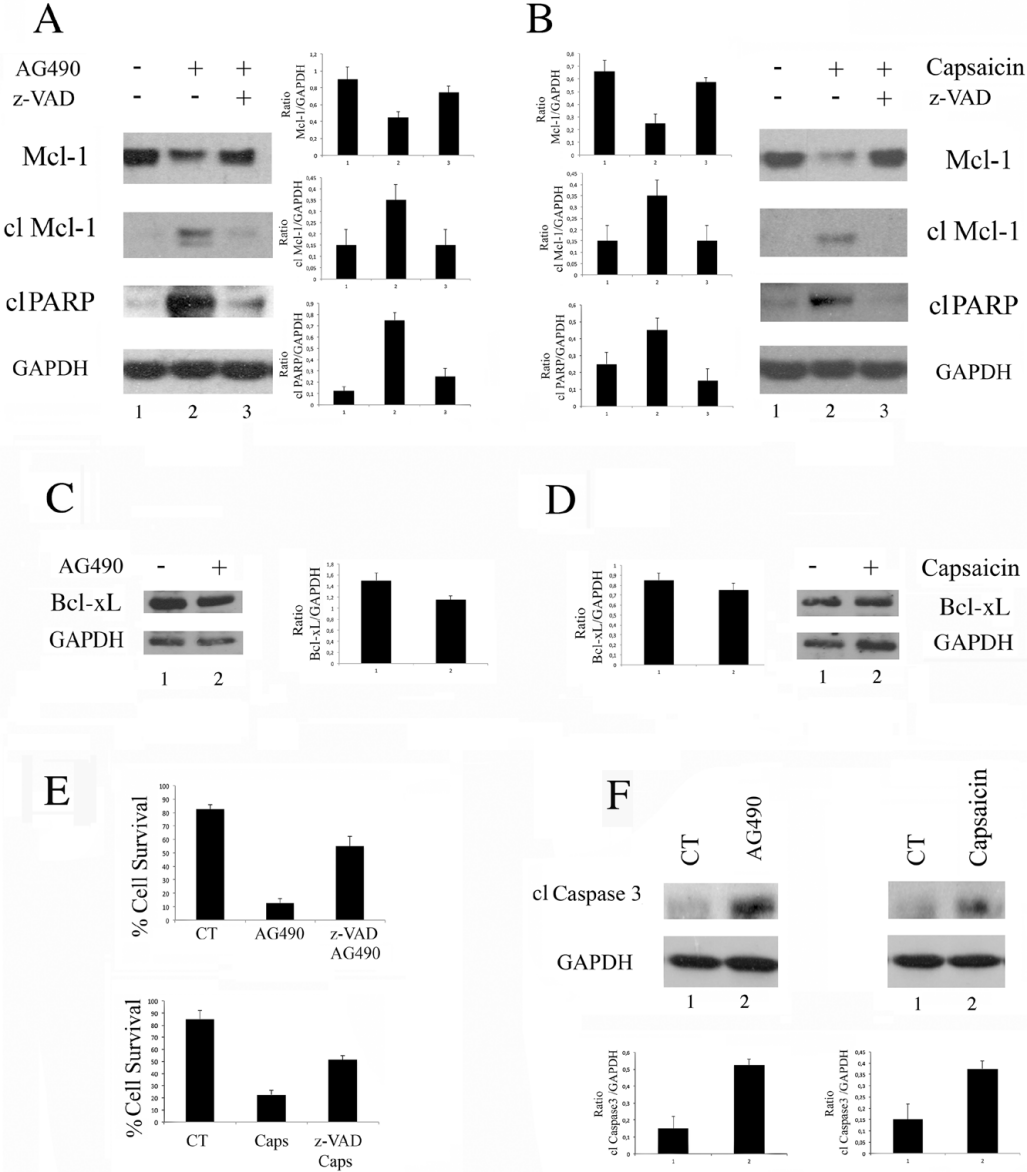
Capsaicin as well as AG490 induces a caspase-dependent cleavage of Mcl-1 and cell death **A.** BC3 cells treated with AG490 STAT3 specific inhibitor (100 μM) or **B.** with Capsaicin (200 μM) were analysed for the expression of Mcl-1 (total and cleaved) or cleaved PARP (cl PARP) by Western blot analysis. GAPDH was included as control. A representative experiment is shown and the mean plus Standar Deviation (SD) of the densitometric analysis of the specific proteins on GAPDH of three independent experiments is also reported; **C.** BC3 cells treated with AG490 STAT3 specific inhibitor (100 μM) or **D.** with Capsaicin (200 μM) were analysed for the expression of Bcl-xL by Western blot analysis. GAPDH was included as control. A representative experiment is shown and the mean plus Standar Deviation (SD) of the densitometric analysis of the specific protein on GAPDH of three independent experiments is also reported; **E.** Percentage of cell survival of BC3 cells treated with AG490 or with Capsaicin (Caps) in the presence or in the absence of Z-VAD-fmk (z-VAD) (50 μM). Mean of the percentage of positive cells plus SD of three independent experiments is also shown; **F.** Caspase3 cleavage was evaluated in BC3 cells treated with AG490 or with Capsaicin, at the above reported dose. GAPDH was included as control. A representative experiment is shown and the mean plus Standard Deviation (SD) of the densitometric analysis of the specific protein on GAPDH of three independent experiments is also reported.

To further demonstrate that Capsaicin mediated cell death correlated with STAT3 de-phosphorylation, we compared its effect with the effect mediated by AG490 STAT3 inhibitor. Since STAT3 inhibition has been previously reported to reduce the expression of the anti-apoptotic molecule Mcl-1, [28] its expression level was evaluated in BC3 PEL cells treated with Capsaicin or AG490. We found that both treatments, besides reducing the expression level of Mcl-1, induced the appearance of its cleaved pro-apoptotic fragment [29] (Figure 3A and 3B). Concomitantly, an increase of the PARP-cleavage (Figure 3A and 3B) was observed in BC3 cells treated with Capsaicin as well as with AG490. We then investigated if, besides Mcl-1, another member belonging to the same family, such as Bcl-xL, would be affected by Capsaicin and/or AG490 and found that it was slightly reduced by both treatments (Figure 3C and 3D). These results indicate that the reduction of Mcl-1 was a rather specific effect correlated with STAT3 de-phosphorylation. Finally, we found that the reduction of cell survival mediated by Capsaicin as well as by AG490 was reverted by z-VAD-fmk pan-caspase inhibitor (Figure 3E), according to the caspase3 activation by both treatments (Figure 3F).

### Capsaicin induced a pro-survival autophagy in PEL cells

**Figure 4:**
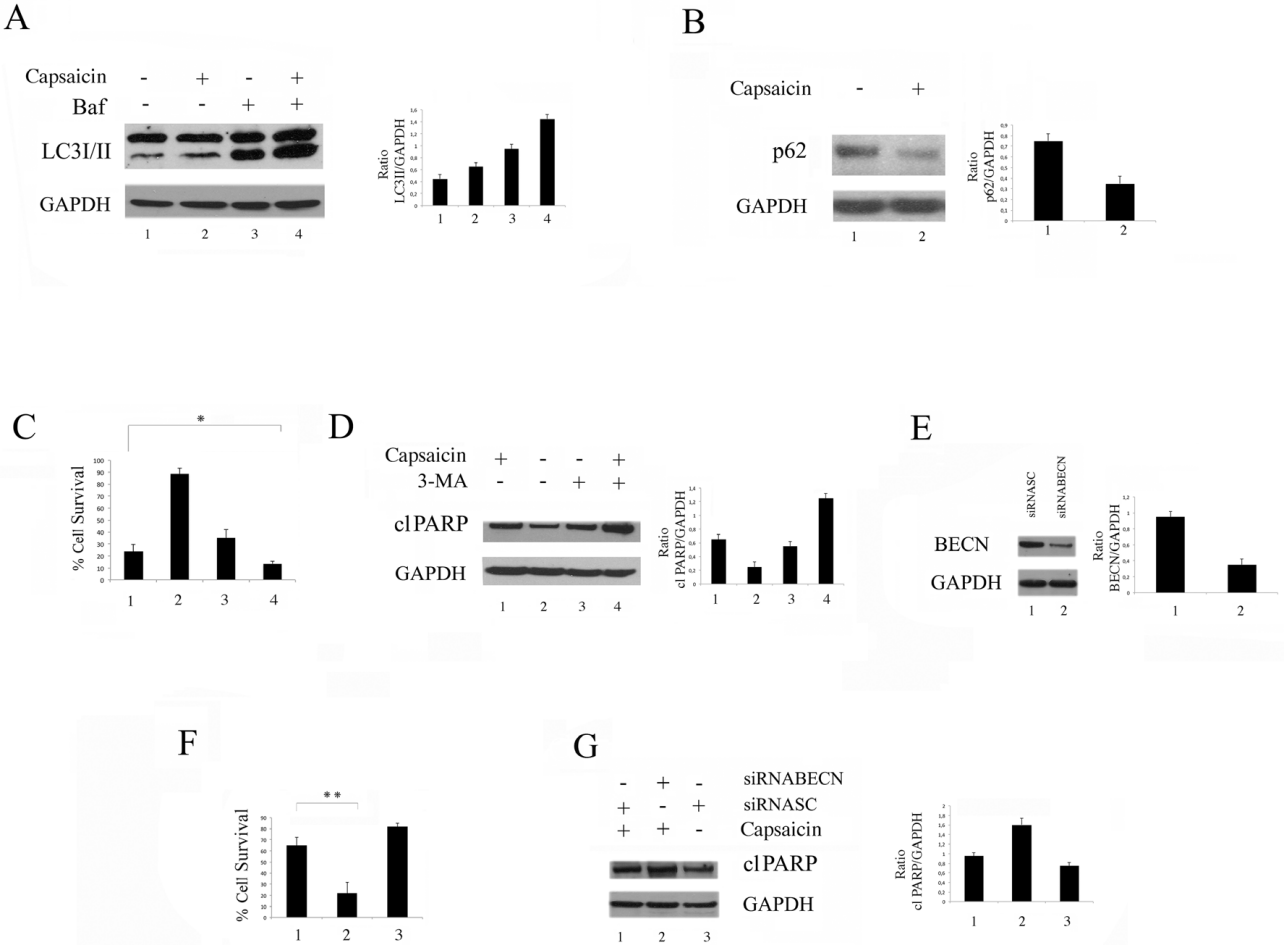
Capsaicin induces a complete and pro-survival autophagy in PEL cells BCBL1 cells were treated with Capsaicin and **A.** the expression of the autofagic marker LC3I/II was analysed in the presence or in the absence of Bafilomycin (Baf) by western blot; **B.** the expression of the autofagic marker p62 was analysed by western blot; GAPDH was included as control and a representative experiment out of three is shown. Mean plus SD of the densitometric analysis of the specific proteins on GAPDH of three independent experiments is also reported; **C.** Percentage of BCBL1 cell survival of cells treated with Capsaicin in the presence or in the absence of 3-MA (5 mM). Mean of the percentage of positive cells plus SD of three independent experiments is also shown. **D.** PARP cleavage (cl PARP). in BCBL1 cells treated with Capsaicin in the presence or in the absence of 3-MA. GAPDH was included as control and a representative experiment out of three is shown. Mean plus SD of the densitometric analysis of the specific protein on GAPDH of three independent experiments is also reported; **E.** BCBL1 cells were scramble treated or silenced for Beclin1 (BECN) and **F.** the percentage of cell survival of PEL cells scramble or silenced for Beclin1 (BECN) after Capsaicin treatment is reported. Mean of the percentage of positive cells plus SD of three independent experiments is shown; *p = 0.02; **p = 0.03. **G.** PARP cleavage (cl PARP) in BCBL1 cells scramble or silenced for Beclin 1 and treated with Capsaicin. GAPDH was included as control and a representative experiment out of three is shown. Mean plus SD of the densitometric analysis of the specific protein on GAPDH of three independent experiments is also reported.

Mcl-1, besides having an anti-apoptotic role, also inhibits autophagy by interacting with Beclin1 [18, 30]. Thus, we investigated if, in correlation to the Mcl-1 reduction, Capsaicin would lead to autophagy induction in PEL cells. At this aim, the main autophagic markers LC3I/II and p62 were analyzed by western blot. As shown in Figure 4A, the lipidated form LC3 (LC3II) accumulated in BCBL1 cells treated with Capsaicin in the presence of Bafilomycin (Baf), an inhibitor of ATP vacuolase that, by blocking LC3II degradation, allows to evaluate LC3 formation and consequently the completeness of the autophagic flux [31]. Conversely, we found that p62 decreased in cells treated with Capsaicin (Figure 4B), further indicating that it was able to promote a complete autophagy in PEL cells. Next, the role of autophagy induced by Capsaicin in PEL cell survival was investigated by inhibiting it with 3-MA or by silencing Beclin 1, an essential autophagic gene. The results obtained, showing that BCBL1 cell survival was reduced (Figure 4C) and PARP cleavage increased in cells pre-treated with 3-MA (Figure 4D) or knockdown for Beclin 1 before Capsaicin treatment (Figure 4E, 4F and 4G), indicate that autophagy played a pro-survival role in PEL cells and that its inhibition could be exploited to increase Capsaicin cytotoxicity against these cells.

### Capsaicin activates monocyte-derived dendritic cells

**Figure 5:**
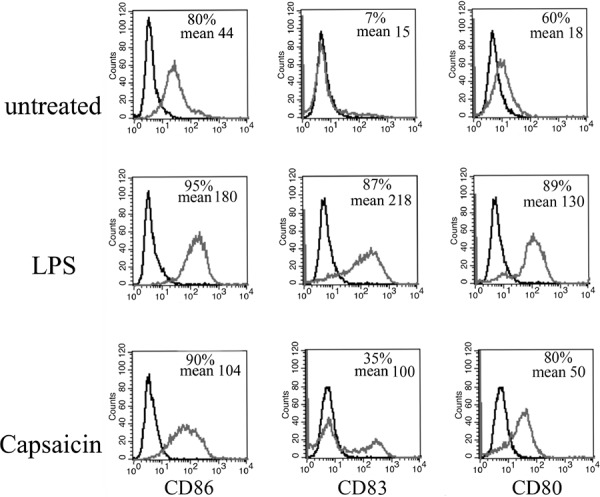
Capsaicin activates DCs DCs were treated with Capsaicin (150 μM) or LPS (100 ng/ml) for 24 hrs and CD86, CD83 and CD80 expression was evaluated by Flow cytometry analysis. Mean fluorescence intensity and the percentage of positive cells is also reported.

Chemotherapies are not able to completely eradicate a tumor if they are not able to activate the immune system [32]. Even if Capsaicin was found to be able to induce in PEL cells the exposure of HSP90 and Calreticulin, that in turn may indirectly lead to DC activation (Figure 1E), we next investigated the effect of Capsaicin on the DCs. At this aim, immature DCs, obtained from monocytes after 6 days of *in vitro* differentiation were left untreated or were exposed to Capsaicin (150 μM) for 24 hours, before analysing the expression of the DC activation markers. As positive control of DC activation, cells were treated with LPS (100 ng/ml) for the same time. The results shown in Figure 5 indicate that Capsaicin up-regulated the expression of the activation and differentiation markers CD86, CD80 and CD83, as evidenced by FACS analysis. The results obtained strongly encourage the use of Capsaicin as chemotherapeutic agent. These results are in agreement with a previous study DCs reporting that Capsaicin activated DCs through the vanilloid receptor1 [21].

### Capsaicin counteracts the immune-suppressive effects on DCs mediated by PEL-conditioned medium

**Figure 6:**
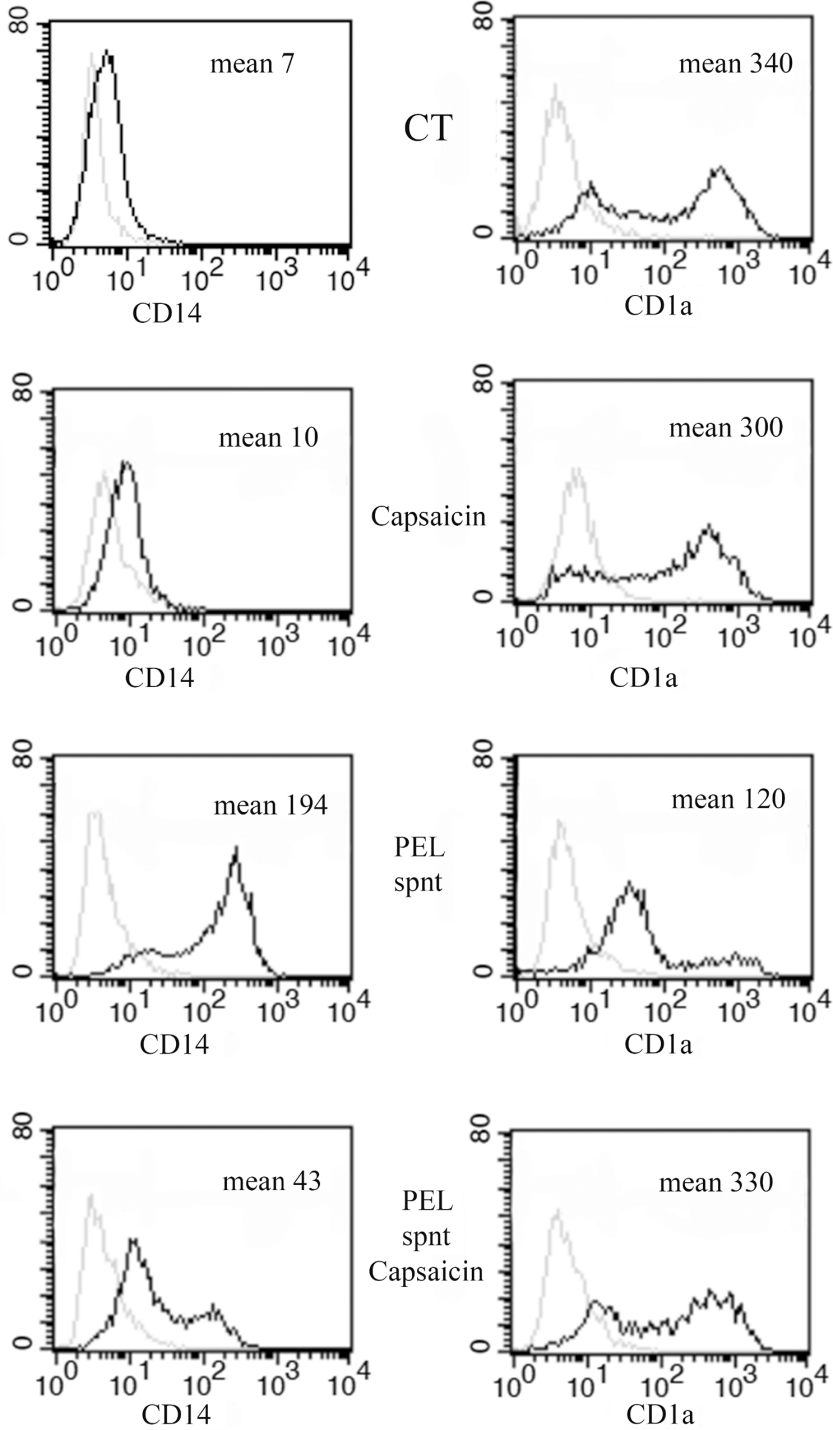
Capsaicin counteracts the inhibition of monocyte-differentiation induced by PEL released factors Surface expression of CD14 and CD1a on immature DCs after 5 days of *in-vitro* differentiation in the presence or in the absence of PEL supernatant with or without Capsaicin (150 μM). One representative experiments out of three is shown. Mean fluorescence intensity is also reported.

Finally, we asked if Capsaicin would be able to counteract the inhibitory effect mediated by PEL supernatant on monocyte differentiation into DCs, previously observed [33]. At this purpose, monocytes, isolated from healthy donors, were cultured with GM-CSF and IL-4 for five days with or without 20% of PEL supernatant in the presence or in the absence of Capsaicin. We found that CD1a expression, strongly reduced by the PEL released factors, present in the PEL cell conditioned medium, was restored in the presence of Capsaicin (Figure 6). Conversely, CD14, retained by PEL supernatant, was down-regulated by the Capsaicin treatment (Figure 6). These results suggest that Capsaicin has the potential to counteract the inhibitory effect on monocytes differentiation mediated by the PEL released factors.

## DISCUSSION

Capsaicin has been successfully employed against a number of different pathological conditions [34] that go from its topic use *in vivo* for pain relief [35] to the anti-cancer effect against several cancer types, either *in vitro* or *in vivo* [2]. STAT3 pathway, inhibited by Capsaicin treatment, induces cell death in cancer cells resistant to conventional chemotherapies, as for example MM and pancreatic cancer [7, 36]. PEL is an aggressive B cell lymphoma associated with KSHV, characterized by a low response to conventional chemotherapies. It displays several similarities with MM, being both characterized by a high level of protein synthesis, cytokine release and by the reliance on the constitutive activation of several pro-survival pathways, including STAT3 [12–14, 37–39]. STAT3 activation depends on the production of cytokines such as IL-6 produced by the tumor itself or encoded by KSHV, [40] whose infection plays a pivotal role in the pathogenesis of this lymphoma [41]. In this study, we found that Capsaicin strongly reduced STAT3 phosphorylation leading to an apoptotic PEL cell death and that its effect was similar to AG490 STAT3 specific inhibitor. STAT3 inhibition by Capsaicin correlates with its pro-death effect, as evidenced by the reversion of STAT3 phosphorylation and the rescue of cell survival by OV, a tyrosine phosphatase inhibitor. Moreover we found that Capsaicin as well as AG490 caused the reduction of the full length Mcl-1 molecule and induced the appearance of its apoptotic cleaved fragment. Mcl-1 belongs to Bcl2 family proteins that, besides having anti-apoptotic functions [28] is also involved in autophagy inhibition by interacting with Beclin1 [30]. Mcl-1 reduction by Capsaicin could be one of the underlying mechanisms leading to both induction of apoptosis and autophagy in PEL cells. The evidence that autophagy inhibition potentiated the Capsaicin-mediated cytotoxic effect against PEL cells suggests that this strategy could be exploited in PEL anti-cancer therapy. It has clearly established that chemotherapies, even the most efficient in the induction of cell death, are not able to completely eradicate a tumor if they do not concomitantly stimulate the cooperation of the immune system and/or counteract the tumor-mediated immune suppression [25, 42]. The finding that Capsaicin induced an immunogenic cell death, in agreement with previous studies obtained in other tumor cells, [43, 44] that Capsaicin directly activated DC and, more importantly, that it counteracted the impairment of monocyte differentiation into DC induced by the PEL released factors, [26] strongly encourage its use in PEL anticancer therapy. In conclusion, we found that at least four goals can be achieved by the use of Capsaicin: 1) to exert a strong cytotoxic effect on PEL cells, that could be further increased by autophagy inhibition, 2) to induce the exposure DAMPs, suggesting the ability to induce an immunogenic cell death 3) to directly activate DCs and 4) last, but not least, to inhibit the immune suppression induced by the PEL released factors. All together, the results obtained in this study, together with the consideration that Capsaicin is a natural product, indicate that Capsaicin could be a possible candidate for an ideal anticancer therapy against PEL as well as other cancers with similar characteristics.

## MATERIALS AND METHODS

### Cells

The BC3 and BCBL1 cells (ATCC), human B-cell lines derived from PEL, carrying latent KSHV, were cultured in RPMI 1640 (Sigma, R0883), 10% Fetal Bovine Serum (FBS) (Euroclone, ECLS0180L), L-glutamine and streptomycin (100 μg/ml) and penicillin (100 U/ml) (Gibco, 10378-016) in 5% CO_2_ at 37°C.

### Cell treatments

BC3 and BCBL1 cells were treated with Capsaicin (Sigma Aldrich, 1091108) at the indicated doses or with Tyrphostin AG490 (100 μM), a Janus JAK2/STAT3 inhibitor (Calbiochem, 658411) for 24 hrs. To study the effect of these chemicals on cell survival, these cells were pre-treated with the pan-caspase inihibitor z-VAD.fmk (50 μM) (Calciochem, 219011) for 30 minutes and then cultured in the presence of Capsaicin (200 μM) (Sigma Aldrich, 1091108) or AG490 (100 μM) (Calbiochem, 658411) for 24 hrs. Similarly, these tumor cells were also pre-treated with Sodium Orthovanadate (OV) (100 μM) (Sigma Aldrich, 450243) for 30 minutes before the addition of Capsaicin (200 μM) (Sigma Aldrich, 1091108) in the culture medium for the following 24 hrs. In order to investigate autophagy, PEL cells were pre-treated with 3-methyladenine (3-MA) (5 mM) (Santa Cruz Biotechnology Inc., sc-205596) for 30 minutes and subsequently treated with Capsaicin (Sigma Aldrich, 1091108) at the indicated doses for 24 hrs. To better elucidate the autophagic mechanism, PEL were cultured with Capsaicin (200 μM) (Sigma Aldrich, 1091108) for 24 hrs and finally treated with Bafilomycin A1 (Baf), an inhibitor of vacuolar-H^+^-ATPase, (20nM) (Santa Cruz Biotechnology Inc., sc-201550) for the last two hours.

### Antibodies

In this work we used the following primary antibodies: mouse monoclonal anti-STAT3 (1:1000) (BD Transduction Laboratories, 610189), mouse monoclonal anti-phosphoSTAT3 (1:100) (pY705) (BD Transduction Laboratories, 612356), rabbit polyclonal anti-PARP p85 Fragment pAb (1:500) (Promega, G7341), rabbit polyclonal anti-Mcl-1 (1:500) (Cell Signaling, 5453P) and goat polyclonal anti-caspase 3 (Santa Cruz Biotechnology Inc., sc-1225). To study autophagy we used the following primary antibodies: rabbit polyclonal anti-LC3 (1:1000) (Novus Biologicals, NB100-2220SS), mouse monoclonal anti-p62 (1:1000) (BD Transduction Laboratories, 610832) and rabbit polyclonal anti-Beclin1 (1:1000) (Cell Signaling, 3738S).

Monoclonal mouse anti-β-actin (1:10000) or anti-GAPDH (1:1000) (Santa Cruz Biotechnology Inc., sc-137179) were used as markers of equal loading” in “were used as markers of equal loading. Goat polyclonal anti-mouse IgG-horseradish peroxidase (HRP) (Santa Cruz Biotechnology Inc., sc-2005) rabbit polyclonal anti-goat IgG-HRP (Santa Cruz Biotechnology Inc., sc-2768) and rabbit IgG-HRP (Santa Cruz Biotechnology Inc., sc-2004) were used as secondary antibodies. All the primary and secondary antibodies used in this study were diluted in a PBS- 0.1% Tween 20 solution containing 3% BSA.

### Western blot analysis

Cells (1 × 10^6^) were washed twice with PBS solution and centrifuged at 1500 rpm for 5 minutes. The pellet was lysed in a RIPA buffer containing 150 mM NaCl, 1% NP-40, 50 mM Tris-HCl (pH 8), 0.5% deoxycholic acid, 0.1% SDS, protease and phosphatase inhibitors. Then, 30 μg of protein lysates were subjected to electrophoresis on 4–12% NuPage Bis-Tris gels (Life Technologies, N00322BOX) according to the manufacturer's instruction. To evaluate the LC3I/II, the cell lysates were denatured in 3X concentrated loading protein buffer (150 mM Tris (pH 6.8), 6% SDS, 30% Glycerol, 30 mM EDTA, 0.2 Bromophenol Blue) for 5 min at 100°C and run on 15% gel (30% acrylamide/Bis Solution 29:1) (Biorad, 161-0159) in Tris-Glycine-SDS buffer. Then, the gels were transferred to Nitrocellulose Membranes (Biorad, Hercules, 162-0115) for 2 hrs in Tris-Glycine. The membranes were blocked in PBS-0.1% Tween20 solution containing 3% of BSA, probed with specific antibodies and developed using ECL Blotting Substrate (Advansta, K-12045-D20).

### Knockdown of beclin 1 by small interfering RNA (siRNA)

The knockdown of Beclin 1 (Santa Cruz Biotechnology Inc., sc-29797) was performed in PEL cell lines using specific small interfering RNA. The day before transfection, 3×10^5^ cells were seeded in 12 well culture plate in RPMI medium without antibiotics. Subsequently, 75 pmoli of siRNA duplex and 7,5 μl of Lipofectamine 2000 Transfection Reagent (Life Technologies, 11668027) were diluted in Optimem medium (Life Technologies, 31985062) and added to the cells for 48 hrs. The transfection efficiency was evaluated by a Fluorescein Conjugate-A siRNA (Santa Cruz Biotechnology Inc., sc-36869), that it was also used as scrambled control.

### Immature DC generation

Human peripheral blood mononuclear cells from healthy donors were isolated by Lympholyte cell separation media (Cedarlane, CL5020). Monocytes were isolated by immunomagnetic cell separation using anti-CD14-conjugated microbeads (Miltenyi Biotec, 1300-50-201). To induce the differentiation of immature DCs (iDCs), monocytes were cultured for 6 days with recombinant human granulocyte-macrophage colony stimulating factor (GM-CSF)(50 ng/mL) (Milteny Biotec, 130-093-865) and interleukin-4 (IL-4) (20 ng/mL) (Miltenyi Biotec, 130-095-373).

### Cell viability

BC3 and BCBL1 cells were plated in 12-well plates at a density of 5 × 10^5^ cells/ml and treated with capsaicin (200 μM) (Sigma Aldrich, 1091108) or with AG490 (100 μM) for 24 hrs. Similarly, PEL cells were also pretreated with Sodium Orthovanadate (OV) (100 μM) (Sigma Aldrich, 450243) for 30 minutes and subsequently cultured in medium containing capsaicin (200 μM) (Sigma Aldrich, 1091108) for 24 hrs.

To also evaluate the impact of autophagy on cell survival, a siRNA assay was performed. PEL cells were cultured in 12-well plates at a density of 3 × 10^5^ cells/ml in RPMI supplemented with 10% FBS without antibiotics. These cells, Beclin 1 knocked-down, were then treated with Capsaicin (200 μM) (Sigma Aldrich, 1091108) for 24 hrs.

After each chemical treatment or RNA interference, a trypan blue (Euroclone, 72571) exclusion assay was performed to test cell viability. Live cells were counted by light microscopy using a Neubauer hemocytometer.

### Apoptosis assay

Treated and untreated cells (5 × 10^5^) were washed in PBS 1X and resuspended in 300 μl hypotonic fluorochrome solution [50 μg/ml propidium iodide, 0.1% sodium citrate, 0.1% Triton-X-100 and 100 U/ml RNase A (all from Sigma) for 30 min at room temperature. DNA content was measured by a FACSCalibur flow cytometer (Becton Dickinson). The mean frequencies of apoptotic subG1 cells were calculated at least from three independent experiments. The percentage of specific apoptosis was calculated as follows: % specific apoptosis = 100 × (% PI^+^ cells − % spontaneous PI^+^ untreated cells)/(100 − % spontaneous PI^+^ untreated cells).

### Immunofluorescence

PEL cells were treated with Capsaicin (200 μM) and the expression of surface calreticulin and HSP90 was analyzed 12 hrs later by flow cytometry using the specific mAbs: anti-Calreticulin (Thermo Scientific, PA3-900) and anti-HSP90 (Santa Cruz Biotechnology Inc., sc-59577).

The expression of surface CD86, CD83 and CD80 was analyzed in untreated and Capsaicin (150 μM) or LPS (100 ng/ml, Sigma) treated immature DC cells (5 × 10^5^) using the following human conjugated antibodies: CD86 (Milteny Biotec, 130-094-878), CD83 (Milteny Biotec, 130-094-181), CD80 (Milteny Biotec, 130-097-202), and the isotype control antibodies (Milteny Biotec, 130-092-213 and 130-092-212).

To study the role of Capsaicin in monocyte differentiation into DCs in the presence or absence of the inhibitory PEL cell supernatant, the expression of surface CD14 and CD1a was analyzed using human mAb anti-CD14 (Milteny Biotec., 130-080-701) and mAb anti-CD1a (BD Pharmingen, 555807)

### Densitometric analysis

The quantification of proteins bands was performed by densitometric analysis using the Image J software, which it was downloaded from NIH web site (http://imagej.nih.gov).

### Statistical analysis

All data are represented by the mean ± standard Deviation of at least three independent experiments.

Student's *t*-test was used for statistical significance of the differences between treatment groups. Statistical analysis was performed using analysis of variance at 5% (*P* < 0.05).

## Abbreviations

Capsaicin: Trans-8-methyl-N-vanillyl-6-nonenamide
STAT3: Signal Transducer and Activator of Transcription 3 (gene)
HSP90: Heat Shock Protein 90
Mcl-1: Myeloid Cell Leukemia 1
DAMPs: Damage Associated Molecular Patterns
VR1: vanilloid receptor 1
GM-CFS: Granulocyte-macrophage colony-stimulating factor
IL-4: Interleukin 4
OV: Sodium Orthovanadate
LC3: Microtubule-Associated protein 1 light chain 3
PEL: Primary Effusion Lymphoma
KSHV: Kaposi's Sarcoma-associated Herpesvirus.

## References

[1] Luo XJ, Peng J, Li YJ (2011). Recent advances in the study on capsaicinoids and capsinoids. Eur J Pharmacol.

[2] Diaz-Laviada I, Rodriguez-Henche N (2014). The potential antitumor effects of capsaicin. Prog Drug Res.

[3] Jung MY, Kang HJ, Moon A (2001). Capsaicin-induced apoptosis in SK-Hep-1 hepatocarcinoma cells involves Bcl-2 downregulation and caspase-3 activation. Cancer Lett.

[4] Lee SH, Krisanapun C, Baek SJ (2010). NSAID-activated gene-1 as a molecular target for capsaicin-induced apoptosis through a novel molecular mechanism involving GSK3beta, C/EBPbeta and ATF3. Carcinogenesis.

[5] Yang ZH, Wang XH, Wang HP, Hu LQ, Zheng XM, Li SW (2010). Capsaicin mediates cell death in bladder cancer T24 cells through reactive oxygen species production and mitochondrial depolarization. Urology.

[6] Ito K, Nakazato T, Yamato K, Miyakawa Y, Yamada T, Hozumi N (2004). Induction of apoptosis in leukemic cells by homovanillic acid derivative, capsaicin, through oxidative stress: implication of phosphorylation of p53 at Ser-15 residue by reactive oxygen species. Cancer Res.

[7] Bhutani M, Pathak AK, Nair AS, Kunnumakkara AB, Guha S, Sethi G (2007). Capsaicin is a novel blocker of constitutive and interleukin-6-inducible STAT3 activation. Clin Cancer Res.

[8] Spitzner M, Ebner R, Wolff HA, Ghadimi BM, Wienands J, Grade M (2014). STAT3: A Novel Molecular Mediator of Resistance to Chemoradiotherapy. Cancers (Basel).

[9] Siveen KS, Sikka S, Surana R, Dai X, Zhang J, Kumar AP (2014). Targeting the STAT3 signaling pathway in cancer: role of synthetic and natural inhibitors. Biochim Biophys Acta.

[10] Bommert K, Bargou RC, Stuhmer T (2006). Signalling and survival pathways in multiple myeloma. Eur J Cancer.

[11] Aoki Y, Feldman GM, Tosato G (2003). Inhibition of STAT3 signaling induces apoptosis and decreases survivin expression in primary effusion lymphoma. Blood.

[12] Cirone M, Di Renzo L, Lotti LV, Conte V, Trivedi P, Santarelli R (2012). Primary effusion lymphoma cell death induced by bortezomib and AG 490 activates dendritic cells through CD91. PloS one.

[13] Gonnella R, Santarelli R, Farina A, Granato M, D'Orazi G, Faggioni A (2013). Kaposi sarcoma associated herpesvirus (KSHV) induces AKT hyperphosphorylation, bortezomib-resistance and GLUT-1 plasma membrane exposure in THP-1 monocytic cell line. Journal of experimental & clinical cancer research : CR.

[14] Granato M, Santarelli R, Gonnella R, Farina A, Trivedi P, Faggioni A (2015). Targeting of prosurvival pathways as therapeutic approaches against primary effusion lymphomas: past, present, and Future. BioMed research international.

[15] Oh SH, Kim YS, Lim SC, Hou YF, Chang IY, You HJ (2008). Dihydrocapsaicin (DHC), a saturated structural analog of capsaicin, induces autophagy in human cancer cells in a catalase-regulated manner. Autophagy.

[16] Choi CH, Jung YK, Oh SH (2010). Autophagy induction by capsaicin in malignant human breast cells is modulated by p38 and extracellular signal-regulated mitogen-activated protein kinases and retards cell death by suppressing endoplasmic reticulum stress-mediated apoptosis. Molecular pharmacology.

[17] Chien CS, Ma KH, Lee HS, Liu PS, Li YH, Huang YS (2013). Dual effect of capsaicin on cell death in human osteosarcoma G292 cells. European journal of pharmacology.

[18] Follin-Arbelet V, Torgersen ML, Naderi EH, Misund K, Sundan A, Blomhoff HK (2013). Death of multiple myeloma cells induced by cAMP-signaling involves downregulation of Mcl-1 via the JAK/STAT pathway. Cancer letters.

[19] Santarelli R, Gonnella R, Di Giovenale G, Cuomo L, Capobianchi A, Granato M (2014). STAT3 activation by KSHV correlates with IL-10, IL-6 and IL-23 release and an autophagic block in dendritic cells. Scientific reports.

[20] Germain M, Slack RS (2011). MCL-1 regulates the balance between autophagy and apoptosis. Autophagy.

[21] Basu S, Srivastava P (2005). Immunological role of neuronal receptor vanilloid receptor 1 expressed on dendritic cells. Proceedings of the National Academy of Sciences of the United States of America.

[22] Beltran J, Ghosh AK, Basu S (2007). Immunotherapy of tumors with neuroimmune ligand capsaicin. Journal of immunology.

[23] Toth BI, Benko S, Szollosi AG, Kovacs L, Rajnavolgyi E, Biro T (2009). Transient receptor potential vanilloid-1 signaling inhibits differentiation and activation of human dendritic cells. FEBS letters.

[24] Hanke N, Alizadeh D, Katsanis E, Larmonier N (2013). Dendritic cell tumor killing activity and its potential applications in cancer immunotherapy. Critical reviews in immunology.

[25] Kroemer G, Galluzzi L, Kepp O, Zitvogel L (2013). Immunogenic cell death in cancer therapy. Annual review of immunology.

[26] Cirone M, Lucania G, Aleandri S, Borgia G, Trivedi P, Cuomo L (2008). Suppression of dendritic cell differentiation through cytokines released by Primary Effusion Lymphoma cells. Immunology letters.

[27] Carballo M, Conde M, El Bekay R, Martin-Nieto J, Camacho MJ, Monteseirin J (1999). Oxidative stress triggers STAT3 tyrosine phosphorylation and nuclear translocation in human lymphocytes. The Journal of biological chemistry.

[28] Thomas LW, Lam C, Edwards SW (2010). Mcl-1, the molecular regulation of protein function. FEBS letters.

[29] Podar K, Gouill SL, Zhang J, Opferman JT, Zorn E, Tai YT (2008). A pivotal role for Mcl-1 in Bortezomib-induced apoptosis. Oncogene.

[30] Decuypere JP, Parys JB, Bultynck G (2012). Regulation of the autophagic bcl-2/beclin 1 interaction. Cells.

[31] Klionsky DJ, Abdalla FC, Abeliovich H, Abraham RT, Acevedo-Arozena A, Adeli K (2012). Guidelines for the use and interpretation of assays for monitoring autophagy. Autophagy.

[32] Green DR, Ferguson T, Zitvogel L, Kroemer G (2009). Immunogenic and tolerogenic cell death. Nature reviews Immunology.

[33] Cirone M, Lucania G, Bergamo P, Trivedi P, Frati L, Faggioni A (2007). Human herpesvirus 8 (HHV-8) inhibits monocyte differentiation into dendritic cells and impairs their immunostimulatory activity. Immunology letters.

[34] Srinivasan K (2015). Biological Activities of Red Pepper (Capsicum annuum) and Its Pungent Principle Capsaicin: A Review. Crit Rev Food Sci Nutr.

[35] Sawynok J (2014). Topical analgesics for neuropathic pain: preclinical exploration, clinical validation, future development. European journal of pain.

[36] Pramanik KC, Fofaria NM, Gupta P, Ranjan A, Kim SH, Srivastava SK (2015). Inhibition of beta-catenin signaling suppresses pancreatic tumor growth by disrupting nuclear beta-catenin/TCF-1 complex: critical role of STAT-3. Oncotarget.

[37] Bharti AC, Donato N, Aggarwal BB (2003). Curcumin (diferuloylmethane) inhibits constitutive and IL-6-inducible STAT3 phosphorylation in human multiple myeloma cells. Journal of immunology.

[38] Granato M, Santarelli R, Lotti LV, Di Renzo L, Gonnella R, Garufi A (2013). JNK and macroautophagy activation by bortezomib has a pro-survival effect in primary effusion lymphoma cells. PloS one.

[39] Sommer VH, Clemmensen OJ, Nielsen O, Wasik M, Lovato P, Brender C (2004). *In vivo* activation of STAT3 in cutaneous T-cell lymphoma. Evidence for an antiapoptotic function of STAT3. Leukemia.

[40] Jones KD, Aoki Y, Chang Y, Moore PS, Yarchoan R, Tosato G (1999). Involvement of interleukin-10 (IL-10) and viral IL-6 in the spontaneous growth of Kaposi's sarcoma herpesvirus-associated infected primary effusion lymphoma cells. Blood.

[41] Punjabi AS, Carroll PA, Chen L, Lagunoff M (2007). Persistent activation of STAT3 by latent Kaposi's sarcoma-associated herpesvirus infection of endothelial cells. Journal of virology.

[42] Cirone M, Di Renzo L, Lotti LV, Conte V, Trivedi P, Santarelli R (2012). Activation of dendritic cells by tumor cell death. Oncoimmunology.

[43] D'Eliseo D, Manzi L, Velotti F (2013). Capsaicin as an inducer of damage-associated molecular patterns (DAMPs) of immunogenic cell death (ICD) in human bladder cancer cells. Cell stress & chaperones.

[44] Gilardini Montani MS, D'Eliseo D, Cirone M, Di Renzo L, Faggioni A, Santoni A (2015). Capsaicin-mediated apoptosis of human bladder cancer cells activates dendritic cells via CD91. Nutrition.

